# Transaortic Shallow Septal Myectomy and Cutting of Secondary Fibrotic Mitral Valve Chordae—A 5-Year Single-Center Experience in the Treatment of Hypertrophic Obstructive Cardiomyopathy

**DOI:** 10.3390/jcm11113083

**Published:** 2022-05-30

**Authors:** Lucian Florin Dorobantu, Toma Andrei Iosifescu, Razvan Ticulescu, Maria Greavu, Maria Alexandrescu, Andrei Dermengiu, Miruna Mihaela Micheu, Monica Trofin

**Affiliations:** 1 Cardiomyopathy Center, Monza Hospital, Tony Bulandra Street 27, 021968 Bucharest, Romania; ludorobantu@gmail.com (L.F.D.); toma.iosifescu@monza.ro (T.A.I.); razvan.ticulescu@monza.ro (R.T.); maria.greavu@monza.ro (M.G.); maria.alexandrescu@monza.ro (M.A.); andrei.dermengiu@monza.ro (A.D.); monica.trofin@monza.ro (M.T.); 2 Department of Cardiology, Clinical Emergency Hospital of Bucharest, Floreasca Street 8, 014461 Bucharest, Romania

**Keywords:** hypertrophic cardiomyopathy, septal myectomy, mitral valve repair, left ventricular outflow tract obstruction

## Abstract

Background: Anomalies of the mitral apparatus have been shown to contribute to left ventricular outflow obstruction in patients with hypertrophic cardiomyopathy (HCM). We report our 5-year single-center experience with a shallow myectomy procedure associated with transaortic mitral valve repair in a cohort of HCM patients. Methods: We studied 83 consecutive patients who underwent surgical treatment of symptomatic left ventricular outflow obstruction. In all study patients, a transaortic shallow septal myectomy was performed. Fibrous or muscular structures connecting the papillary muscles to the septum or free wall were resected, and fibrotic secondary chordae of the anterior mitral valve were cut selectively. Results: We report one death (1.2%) during hospitalization, no iatrogenic ventricular septal defects, and two (2.4%) mitral valve replacements. At discharge, no patients were in New York Heart Association (NYHA) Class III/IV, from 49 (59%) preoperatively. Mean maximal septal thickness decreased from 24 ± 6 to 16 ± 3 mm. Mean outflow gradient decreased from 93 ± 33 to 13 ± 11 mmHg. Grade 3 or 4 mitral regurgitation was noticed in one patient postoperatively, from 32 (39%) before surgery. Conclusions: Shallow septal myectomy associated with secondary mitral valve chordal cutting and papillary muscle mobilization provided excellent results offering adequate treatment of outflow obstruction.

## 1. Introduction

Hypertrophic cardiomyopathy (HCM) is a myocardial genetical disease characterized by left ventricular (LV) hypertrophy, the mechanism of its appearance being still not fully understood [1,2,3]. The prevalence of HCM is approximated to be 1 in 500 people [1,3]. In recent years, the transaortic myectomy emerged as the optimal treatment of the left ventricular outflow tract (LVOT) gradient in patients with HCM. The indication for surgery is well established for patients who experience obstructive symptoms and for whom a higher than 50 mmHg LVOT gradient persists despite optimal medical therapy [1,4]. In comparison with other methods, the concept of septal myectomy focuses on various specific patterns of hypertrophy distribution and also its individual severity. Significant obstruction may also occur in a small subcategory of patients with a rather thin septum (<18 mm) and, in these cases, various anomalies of the mitral apparatus contribute to obstruction [5].

In the current era, offering the best invasive treatment for LVOT obstruction for the growing number of surgical HCM candidates remains a major challenge [6] and is provided by only a small number of referral centers in North America and Europe. We present a new surgical protocol, recently implemented at our center, in which 83 consecutive patients with drug-refractory symptomatic obstructive HCM underwent a shallow myectomy, associated with cutting of the fibrotic secondary anterior mitral valve (MV) chordae and papillary muscle mobilization.

## 2. Materials and Methods

### 2.1. Research Design and Sample

We retrospectively studied a consecutive cohort of 83 patients diagnosed with obstructive HCM, who underwent septal myectomy and transaortic MV repair, performed by a single surgeon and his team, at our center (Monza Hospital, Bucharest, Romania), between June 2015 and May 2020. All the 83 patients studied were consecutive, there were no exclusion criteria. All study patients had a resting or provoked LVOT gradient ≥ 50 mmHg and important drug-refractory symptoms. The medical therapy used consists of beta blockers and calcium channel blockers in the maximal-tolerated doses, for at least 6 months before surgery. A comparison of clinical and hemodynamic status (by echocardiography) before surgery and at hospital discharge was made. A full preoperative informed consent was obtained from all patients before surgery. All patients underwent preoperative transthoracic echocardiography (TTE), coronary angiogram, left ventriculography, cardiac magnetic resonance imaging (CMR), and intraoperative transesophageal echocardiography (TEE). Based on the preoperative findings, we planned in each patient a myectomy of about one third of the interventricular septum (IVS) thickness and a transaortic MV repair, consisting in cutting fibrotic secondary chordae of the anterior MV and papillary muscle mobilization.

Associated procedures were: coronary artery bypass grafting (CABG) (9 patients), aortic valve repair (4 patients), aortic valve replacement (2 patients), tricuspid valve repair (2 patients), and atrial septal defect closure (2 patients).

### 2.2. Surgical Procedure

After induction of general anesthesia, TEE was routinely performed in order to further determine the extent of septal myectomy and assess MV morphology as well as the presence of associated anomalies of the mitral apparatus. Full sternotomy and standard cannulation were used. After aortic cross-clamping, antegrade cold blood cardioplegia with esmolol was infused, with subsequent doses not more than 20 min apart.

**Figure 1:**
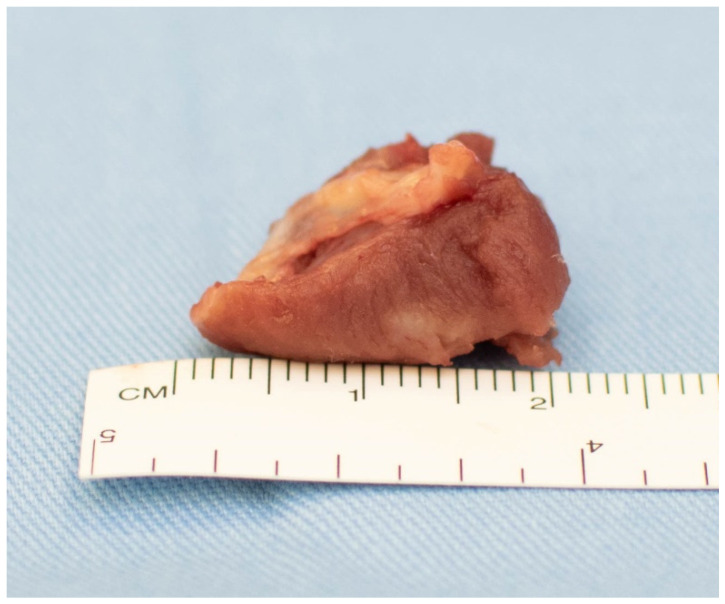
Septal myectomy piece.

**Figure 2:**
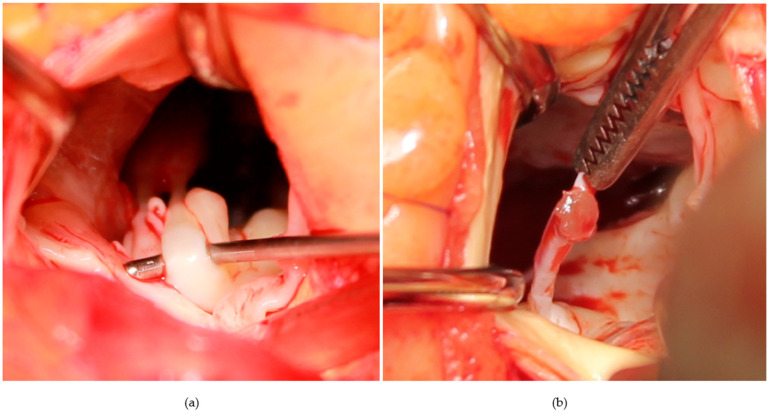
(**a**) Thick secondary chord of the anterior mitral leaflet; (**b**) secondary chord cut at its papillary muscle insertion; it is still attached to the anterior mitral leaflet.

**Figure 3:**
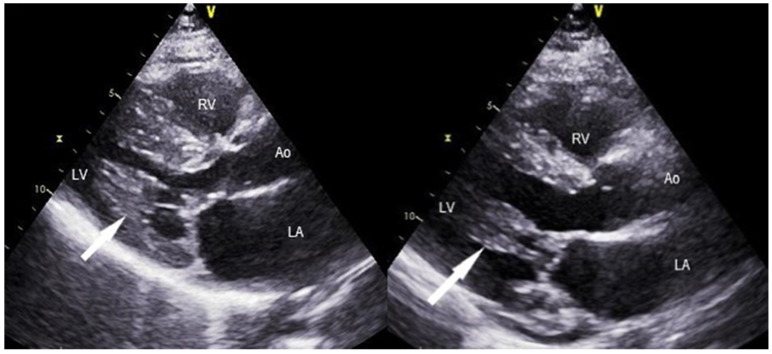
Preoperative (**left**) and postoperative (**right**) TTE showing the pathophysiological substrate for papillary muscle mobilization and secondary MV chordal cutting associated with septal myectomy in obstructive HCM. The arrow indicates a papillary muscle that has been freed surgically from its aberrant attachment to the LV free wall. In addition, a significant enlargement of the LVOT is noticed after surgery, along with posterior displacement of the mitral coaptation point.

In all study patients, through an aortotomy, only a shallow septal myectomy was performed, encompassing about one third of the septal thickness. Great care was taken to ensure that the muscular excision went beyond the point of systolic mitral-septal contact [5,7] and beyond the equator of the LV, if a mid-ventricular stenosis was noticed preoperatively. Another key point of this step is to remove the muscle in a single piece [5,7], preventing fragment embolization (as illustrated in Figure 1). The mean mass of resected muscle in our patients was 4.5 g (range 1 to 12 g). The ventricular surface of the anterior MV leaflet was systematically analyzed. Fibrotic and retracted secondary chordae of the anterior MV leaflet were examined and individually selected before being cut (mean number of cut chordae was 6, with a range from 2 to 13) (Figure 2). Great consideration was given to identifying fibrous or muscular structures that aberrantly connected the papillary muscles to the septum or free wall (Figure 3) and also resected, in order to increase papillary muscle mobility [5,7,8]. These procedures have the role of reducing tethering of the anterior MV leaflet, thus displacing the mitral apparatus posteriorly and away from the LVOT. Elongation of mitral leaflets is probably the most frequent anomaly associated with obstructive HCM. Occasionally, after excision of the retracted secondary chordae of the anterior mitral leaflet, elongated (slack) primary chordae can be observed. These usually pertain to the central part of A2, one from each papillary muscle. Both anomalies can be corrected by transversely plicating the free margin of A2 between the redundant chordae. We used this technique in all 15 patients (18%) presenting these abnormalities. No other mitral valve repair techniques were used.

After weaning from cardiopulmonary bypass, TEE was repeated to assess the relief of obstruction as well as MV competence.

### 2.3. Statistical Analysis

Data were reported as proportions or mean ± standard deviation. The preoperative and postoperative values were analyzed using the Student *t*-test, chi-square test or Fisher exact test, as necessary. A *p*-value ≤ 0.05 was interpreted as statistically significant.

## 3. Results

### 3.1. Patients’ Demographic and Preoperative Characteristics

Baseline preoperative characteristics of the 83 study patients are as follows: the age ranged from 18 to 79 years, with a mean of 52 ± 14 years, and 51 (61%) were males. Forty-nine (59%) patients were categorized as New York Heart Association (NYHA) classes III or IV, while the rest (*n* = 34, 41%) were considered as NYHA class II. The mean IVS thickness was 24 ± 6 mm, and the mean LVOT gradient was 93 ± 33 mmHg. Moderate-to-severe or severe mitral regurgitation (grade 3 or 4) was present in 32 (39%) subjects. Twenty-six (31%) patients associated atrial fibrillation (AF).

### 3.2. Postoperative (Predischarge) Data

Postoperative outcome after septal myectomy and MV repair is presented in Table 1.

**Figure 4:**
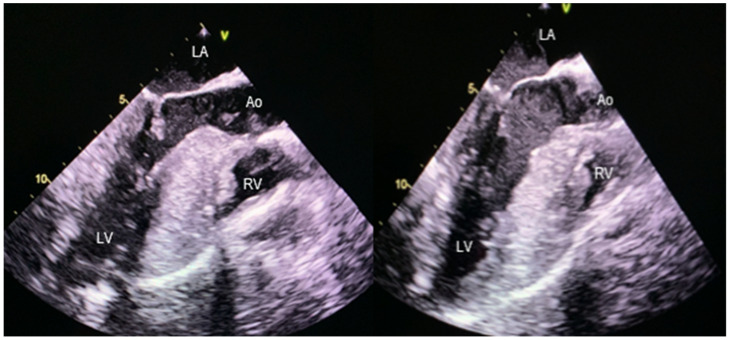
Preoperative (**left**) and postoperative (**right**) TEE showing the immediate results of septal myectomy and MV repair in obstructive HCM. Preoperative systolic anterior motion of the mitral valve is evident. A postoperative widening of the LVOT is noticed, not only by septal excision, but also by posterior relocation of the mitral apparatus.

Examples of echocardiographic outcome after septal myectomy and MV repair using the technique described above are presented in Figure 3 and Figure 4.

## 4. Discussion

The current study presents postoperative clinical and echocardiographic data from 83 consecutive patients with HCM and LVOT obstruction who underwent septal myectomy and MV repair in the Cardiomyopathy Center of Bucharest Monza Hospital. Patients’ demographic data were comparable with statistics reported by Romanian Registry of Hypertrophic Cardiomyopathy [9] or other cohorts of Romanian patients with HCM [10], with a mean age falling in the sixth decade of life, and with male predominance. Baseline clinical characteristics and echocardiography findings were similar with those from other European centres [11,12]. Preoperatively, the majority of our subjects (59%) were either in NYHA class III or IV, with a mean IVS thickness of 24 ± 6 mm and a mean LVOT gradient of 93 ± 33 mmHg. In addition, postoperative outcomes at hospital discharge are in line with those observed in other surgical cohorts, with significant reduction in peak resting/provoked intraventricular gradient and significant improvement in heart failure symptoms [11,13,14]. A postoperative residual gradient of >30 mmHg was noticed in only 5 (6%) patients, consistent with literature data [14,15]. Notably, recent evidence from other groups, as well as our unpublished preliminary data, showed sustained improvement in hemodynamic and functional status during long-term follow-up [16].

Various pathophysiological changes encountered in HCM (such as diastolic dysfunction, MV anomalies and regurgitation, and LVOT obstruction) lead to progressive left atrial remodeling and dilatation, and eventually to AF onset and persistence [17,18,19]. It has been shown that AF has a prevalence of 20–25% in HCM patients, being more frequent in older subjects and in those with LVOT obstruction [20,21,22]. In our study, a lower incidence of AF was reported postoperatively. One might hypothesize that the disruption of the triggering mechanisms secondary to septal reduction and adjunctive MV repair could contribute to decreased arrhythmia. In addition, in the HCM population, the episodes of atrial fibrillation are unpredictable in frequency and timing [23], so long-term follow-up of the study cohort is necessary to detect AF onset/recurrence.

The first-line approach to obstructive HCM is pharmacological—and beta-blocker, non-dihydropiridinic calcium channel blocker as well as disopyramide (when available) should be prescribed in the maximal tolerated doses [4], including emerging molecules such as mavacamten [24,25]. In the majority of cases, obstruction is determined by protrusion of hypertrophied septum, but also by dragging forces of the turbulent blood jet actioned in systole upon an abnormal mitral apparatus [26]. When guideline-directed medical treatment is not sufficient for symptom relief, septal reduction therapy is recommended.

The historical course of surgical septal myectomy starts in 1961 and refers to cutting an important part of the septum through an aortotomy, being until today the standard treatment for patients with significant obstruction despite optimal medical therapy [27,28]. Early data based on large multicenter registries [29,30] showed a relatively high (≥5%) perioperative mortality after septal myectomy, with substantial variability when the hospital procedural volume was taken into account (3.8% in the highest tertile vs. 15.6% in the lowest volume tertile).When performed by experienced operators in comprehensive centers, septal myectomy is a low-risk operation (mortality < 1%) with a clinical success over 90% to 95% [31,32,33,34,35,36]. Indeed, in most cases, this seems to resolve the gradient issue—as proved by the Mayo Clinic experience [37], and the American guidelines recommend septal myectomy for patients with resistant obstruction even since 2011 [1]. In our study, the mortality rate was 1.2% (1 patient). It is to be mentioned that the death occurred in a patient with associated triple coronary bypass surgery. The need for concomitant CABG has been acknowledged as an independent predictor of death since 2005 by Woo and colleagues in a cohort of 338 HCM patients who underwent septal myectomy [38]. These findings were subsequently confirmed in a larger cohort (*n* = 2913) of adult patients with obstructive HCM managed at Mayo Clinic; a significantly increased operative mortality was evidenced in subjects undergoing myectomy plus CABG compared with a control group treated by septal myectomy alone (2.2% vs. 0.8%; *p* = 0.048) [39].

Besides the transaortic approach, there is also transmitral myectomy. This technique enables an all-inclusive view of IVS and MV apparatus and the ability to concomitantly address MV pathology [40]. Although attractive due to aforesaid advantages, the transmitral myectomy has a number of shortcomings which prevent its routine use. Firstly, the exposure of IVS and MV might be impaired by the hypertrophied ventricle and reduced left atrium compliance. Secondly, compared with the standard method (i.e., the transaortic approach), there is an increased risk of injury to the conduction system since the location of the first septal incision is more difficult to judge. Thirdly, increased incidence of perioperative atrial fibrillation might occur subsequent to the left atrium manipulation [41].

MV malformations in obstructive HCM represent a primary phenotypic expression of the disease [42,43,44,45], thus a surgical procedure is strongly advised for those patients with structural abnormalities of the mitral apparatus [27]. It is therefore rational that the mitral apparatus should be thoroughly examined before surgery, using multimodality imaging (TTE, TEE, and CMR) and MV repair should be, in our view, part of the standard surgical protocol.

The cause of mitral regurgitation in obstructive HCM is the poor coaptation of the posterior leaflet to the abnormally anteriorized anterior leaflet, either because of insufficient length or from restricted mobility—this resulting in an eccentric regurgitant jet directed towards the posterior left atrial wall [42]. Other competitive causes of mitral regurgitation (as annulus dilation or degenerative calcification) will divert the flow to a central or multiple directions. In our study, echocardiography (through effective regurgitant orifice area measurement) and CMR (through flow sequence) proved to be useful in quantifying the severity of mitral regurgitation (regurgitant flow and fraction). Thanks to its ideal spatial resolution, CMR had a great role in describing the structural abnormalities of the mitral apparatus, guiding therefore the decision of repair or replacement. Replacement of the MV has already been shown to worsen the postoperative outcome [46] and only 2 patients in our cohort remained true candidates for valve replacement after preoperative evaluation. Our study shows that in many patients, the cause of abnormal tethering of the MV is the presence of fibrotic secondary chordae and resecting them led to an adjusted geometry of the MV with a posteriorly diverted coaptation point, away from the LVOT.

In daily practice, MV surgery as part of septal reduction is still a matter of debate, depending on the morphology of the MV and associated apparatus, and the presence of any intrinsic MV leaflet disease. Adjunctive MV procedures comprise anterior leaflet plication/extension [47,48], reorientation of papillary muscles [49], secondary chordae cutting [5,50,51] and edge-to-edge repair [51,52], aiming to completely alleviate LVOT obstruction by decreasing the mobility of the anterior leaflet and reorient the MV apparatus posteriorly. Our experience suggests that an alternative to the standard procedure, presuming only a shallow myectomy, but associated with cutting of secondary mitral chordae and papillary muscle mobilization can effectively abolish the LVOT gradient. In our study, the only indication for MV replacement was significant MV stenosis. The surgical outcome of patients with obstructive HCM is linked with the experience of the surgeon with the procedure but also by the experience of the center in perioperative management of these cases [30,53]. Considering that our statistics are based on a 5-year-practice, the promising results stand as an argument for a steep learning curve for the surgical practitioner. In our opinion, the addition of procedures on the MV apparatus reduces the need of a deep septal myectomy, a technique associated with significant morbidity in unexperienced centers. When the surgeon is experienced in mitral valve repair surgery, a situation more common nowadays, avoiding the deep septal myectomy by adding a gesture on the mitral valve is, in our opinion, the reason for practically no learning curve in this kind of operation. In recent years, we are witnessing growing referral centers in Europe [7] and, currently, there are data registers of HCM surgical management in Italy, Romania, and Ukraine, adding experience and expertise to dedicated centers as ours.

## 5. Conclusions

Shallow septal myectomy, associated with secondary mitral valve chordal cutting and papillary muscle mobilization provided excellent results. This approach was associated with a particularly favorable clinical outcome. Engaging a multidisciplinary team in planning the surgery proved to bring an added benefit. In our opinion, this novel approach offers adequate treatment of LVOT obstruction to the many and often young patients with obstructive HCM and drug-refractory heart failure symptoms.

## Figures and Tables

**Table 1:** Outcome after septal myectomy and mitral valve repair.

Variable		Preoperative	Postoperative ^a^	*p*-Value
Hospital mortality		-	1 (1.2%)	-
Iatrogenic septal defect		-	0	-
NYHA class III/IV		49 (59%)	0	<0.0001
Atrial fibrillation		26 (31%)	13 (16%)	<0.05
Maximum septal thickness		24 ± 6 mm	16 ± 3 mm	<0.0001
LV outflow gradient		93 ± 33 mmHg	13 ± 11 mmHg	<0.0001
Mitral regurgitation grade	0/1	12 (14%)	52 (65%)	
	2	39 (47%)	27 (34%)	
	3/4	32 (39%)	1 (1.2%)	<0.0001
LV ejection fraction		63 ± 5%	59 ± 5%	<0.0001
Mitral valve replacement		-	2 (2.4%)	-
Mitral valve repair		-	81 (97.6%)	-
Aortic valve repair		-	4 (4.8%)	-
Aortic valve replacement		-	2 (2.4%)	-
Tricuspid valve repair		-	2 (2.4%)	-
Atrial septal defect closure		-	2 (2.4%)	-
Pacemaker implantation		-	8 (9.6%)	-
CABG		-	9 (10.8%)	-

Values are expressed as mean ± standard deviation, or number (percentage). ^a^ Assessed at hospital discharge.
